# *Youngia lushanensis*, a new species of Asteraceae from Sichuan, China, inferred from morphology and nr*ITS* phylogeny

**DOI:** 10.3389/fpls.2024.1453759

**Published:** 2024-12-04

**Authors:** Yulan Peng, Xuemei Pu, Xianhua Xiong, Lijie Cheng

**Affiliations:** ^1^ Chengdu Institute of Biology, Chinese Academy of Sciences, Chengdu, China; ^2^ College of Life Sciences and Technology, Mianyang Tearchers’ College, Mianyang, Sichuan, China; ^3^ College of Ecology and Evironment, Chengdu University of Technology, Chengdu, China

**Keywords:** *Youngia*, Asteraceae, morphology, ITS, new species

## Abstract

*Youngia lushanensis* (Asteraceae), a new species of *Youngia*, is described and illustrated from Lushan in Sichuan, Southwest China. Its systematic position is evaluated based on a molecular phylogenetic analysis of the nuclear ribosomal *ITS* and on morphological comparison with related species. It is morphologically similar to *Y. purpimea* and *Y. szechuanica*, with purplish red abaxially leaves. But it differs from the latter two in that its caudex are glabrous, leaf margins are sparsely dentate, and being glabrous on both surfaces, 9-11 florets in each capitulum, and inner phyllaries are crested. Phylogenetic analysis based on ITS also showed this new species is closely related with *Y. purpimea* and *Y. szechuanica.* The three species *Y*. *lushanensis Y. purpimea* and *Y. szechuanica*, formed a basal group of *Youngia*, which is distributed in moist slopes in Southwest China.

## 1 Introduction

*Youngia* Cassini belongs to the Asteraceae family, Cichorioideae subfamily and Tribe Cichoreae, and a member of euasterids II clade of Asterids (APGIV, 2016). Babcock and Stebbins (1937) subdivided this genus into six sections, i.e. “ Sect. *Crepidiopsis*”, “Sect. *Desiphylum*”*. “*Sect. *Stenophytum*”, Sect. “*Hieraciella*” and Sect. “*Mesomeris*” and “sect. *Euyoungia*”. There is considerable controversy among different scholars regarding the circumscription of *Youngia* (Babcock and Stebbins, 1937; Hu, 1969; Sennikov and Illarionova, 2008; Maity and Maiti, 2010; Shi and Kilian, 2011). Our former study found that the genus *Youngia* of Babcock & Stebbins systematics is not a monophyletic group, *Youngia diversifolia* and *Y. nansiensis* (“Sect. *Crepidiopsis* Babcock & Stebbins”), should be a member of the genus *Crepidiastrum*. *Paraixeris humifusa* (Dunn) C. Shih should be transferred to the genus *Youngia* (Peng et al., 2014). There is confusion in the boundary between *Youngia* and its related genera *Crepidiastrum* Nakai. *Crepidiastrum*, is a genus established by Nakai (1920), with about 16 species: C. and E. Asia, including N. Pacific Bonin (Ogasawara) Islands, nine species (two endemic) in China, including *Crepidifolium* Sennikov, Geblera Kitagawa (1937), and *Paraixeris* Nakai [=*Youngia* Cass. sect. *Paraixeris* (Nakai)] Kitam. (1942) (Shi and Kilian, 2011; Peng et al., 2014). Phylogenic studies showed that *Crepidiastrum* is the sister group of *Youngia* (Kilian et al., 2009; Zhang et al., 2011; Peng et al., 2014).

In addition to the widespread distribution of *Y*. *japonica* L., *Youngia* is a genus consisting of approximately 30 species mainly distributed in East Asia (Shi and Kilian, 2011). China is the diversity center of this genus, with 28 species (22 endemic) in China (Shi and Kilian, 2011). Recently, as botanical research continues, more new species of *Youngia* have been discovered in China. *Youngia zhengyiana* T. Deng, D.G. Zhang, J.W. Zhang & H. Sun was found in Lipo County, Guizhou Province (Deng et al., 2014). Later, three new species *Y*. *purpimea* Y.L. Peng, W.B. Ju, X.F. Gao & Y.D. Gao (Peng et al., 2015), *Y. jiulongensis* Y.L. Peng, X.F. Gao & Li Bing Zhang (Peng et al., 2017), and *Youngia baoxingensis* Y.S. Chen (Chen, 2018) were reported from Sichuan Province. *Youngia gongshanensis* Y.S. Chen & R. Ke was found from Yunnan Province by Ke and Chen (2016). *Youngia jiulongshanensis*. Cai, Y.L. Xu & X.F. Jin was found from south-western Zhejiang Province of East China by Cai et al. (2019). *Youngia hangii* T. Deng, D.G. Zhang, Qun Liu & Z.M. Li from Hubei Province of Central China by Liu et al. (2021). The systematics and diversity of *Youngia* still need to be studied, especially for the biennial or perennial taxa, due to significant morphological variations in flowers and leaves. In 2023, the author collected a unique member of this genus in Lushan County, Sichuan Province, China. In this paper, new materials were collected, morphology evaluation and molecular analyses were employed to determine the phylogeny of *Youngia*.

## 2 Methods and materials

### 2.1 Plant materials and morphological assessment

The plants were collected in Lushan County. A comparative study was conducted on the morphological characteristics of this species with its closely related species. Five species of *Crepidiastrum* and 19 specimens of *Youngia* were sampled. All species of *Youngia* were used as in-groups, and those of *Crepidiastrum* were used as outgroups.

Leaf material for DNA analysis was generally obtained (as silica gel-dried material) from wild populations. All vouchers of samples in our experiments and the holotype specimens of *Y. lushanensis* were deposited in the Herbarium of the Chengdu Institute of Biology (CDBI). In total, 8 out of 33 DNA sequences were newly generated for this study (see **Table 1**). Taxa sampled, voucher information, and GenBank accession numbers for the datasets were listed in **Table 1**. The plant material used for morphological studies were listed in **Supplementary Material S1**. The shape of the leaves, leaf attachment pattern, the morphology of the stems, the number of florets per capitulum, pappus color and whether the inner phyllaries have appendages are important taxonomic features of this genus. Thus, inner phyllaries, basal part of stem, pappus, leaves were chosen for qualitative characters. Florets per capitulum, length of inner phyllaries, pappus and achenes, number of capitula and leaf size were chosen for quantitative characters to study the related species morphology. Indumentum of leaves and petioles, achenes were observed and then were taken photos using a light microscope (Zeiss 2000). T-test method was used to analyzing the significance of differences in quantitative traits between new species and related species.

**Table 1:** Voucher information and GenBank accession numbers for the 25 species of *Crepidiastrum* and *Youngia* genera.

Taxon name	Voucher information	*ITS*
*Youngia japonica*	TENN: Schilling s. n	HQ161935
*Youngia japonica*	Zhang JW 388 (KUN)	HQ436229
*Youngia japonica*	GAT-bg114	AJ633294
*Youngia pseudosenecio*	X.F. Gao, Y.L. Peng, B. Xu & X. Zheng 11645 (CDBI)	PP785561
*Youngia rubida*	L. Peng pl001 (CDBI)	PP789654
*Youngia hangii*	HHE 3256 (KUN)	MZ817057
*Youngia humifusa*	Y.L. Peng & L.J. Tong 13147 (CDBI)	KC968035
*Youngia erythrocarpa*		AB598566
*Youngia heterophylla*	X.F. Gao, Y.L. Peng, B. Xu & X. Zheng 11996 (CDBI)	PP784683
*Youngia gracilipes*	Boufford & al. 34392 (A, KUN)	KJ502309
*Youngia henryi*	Y.L. Peng365 (CDBI)	KR733609
*Youngia pilifera*	X.F. Gao, Y.L. Peng, B. Xu & X. Zheng 11779 (CDBI)	PP784685
*Youngia paleacea*	Boufford & al. 34500 (A, KUN)	KJ502311
*Youngia thunbergiana*	Urbatsch 10460 (LSU)	KC539465
*Youngia zhenyiana*	Deng 2945 (KUN)	KJ502314
*Youngia cineripappa*	ZZ09097 (KUN)	KJ502307
*Youngia cineripappa*	ZZ09041 (KUN)	KJ502306
*Youngia purpimea*	X.F. Gao, Y.L. Peng, B. Xu & X. Zheng11573-2 (CDBI)	PP785562
*Youngia purpimea*	X.F. Gao, Y.L. Peng, B. Xu & X. Zheng11573-1 (CDBI)	PP785564
*Youngia szechuanica*	Zhang 1030 (KUN)	KJ502313
*Youngia lushanensis*	X.H. Xiong 20230602-2 (CDBI)	PP785563
*Youngia lushanensis*	X.H. Xiong 20230602-1 (holotype, CDBI)	PP785560
*Youngia jiulongshanensis*	Y.L. Xu 621	MN701104
*Youngia jiulongshanensis*	Y.L. Xu 627	MN701105
*Youngia longipes*	DengSN1772 (KUN)	PP764805
*Youngia longipes*	SN (KUN)	PP764804
*Youngia longipes*	SN (KUN)	PP764803
*Crepidiastrum ameristophyllum*		AB002610
*Crepidiastrum linguaefolium*		AB002602
*Crepidiastrum grandicollum*		AB002596
*Crepidiastrum taiwanianum*		AB002615
*Crepidiastrum sonchifolium*	KUS-F19490	KT249900

### 2.2 Study area

**Figure 1:**
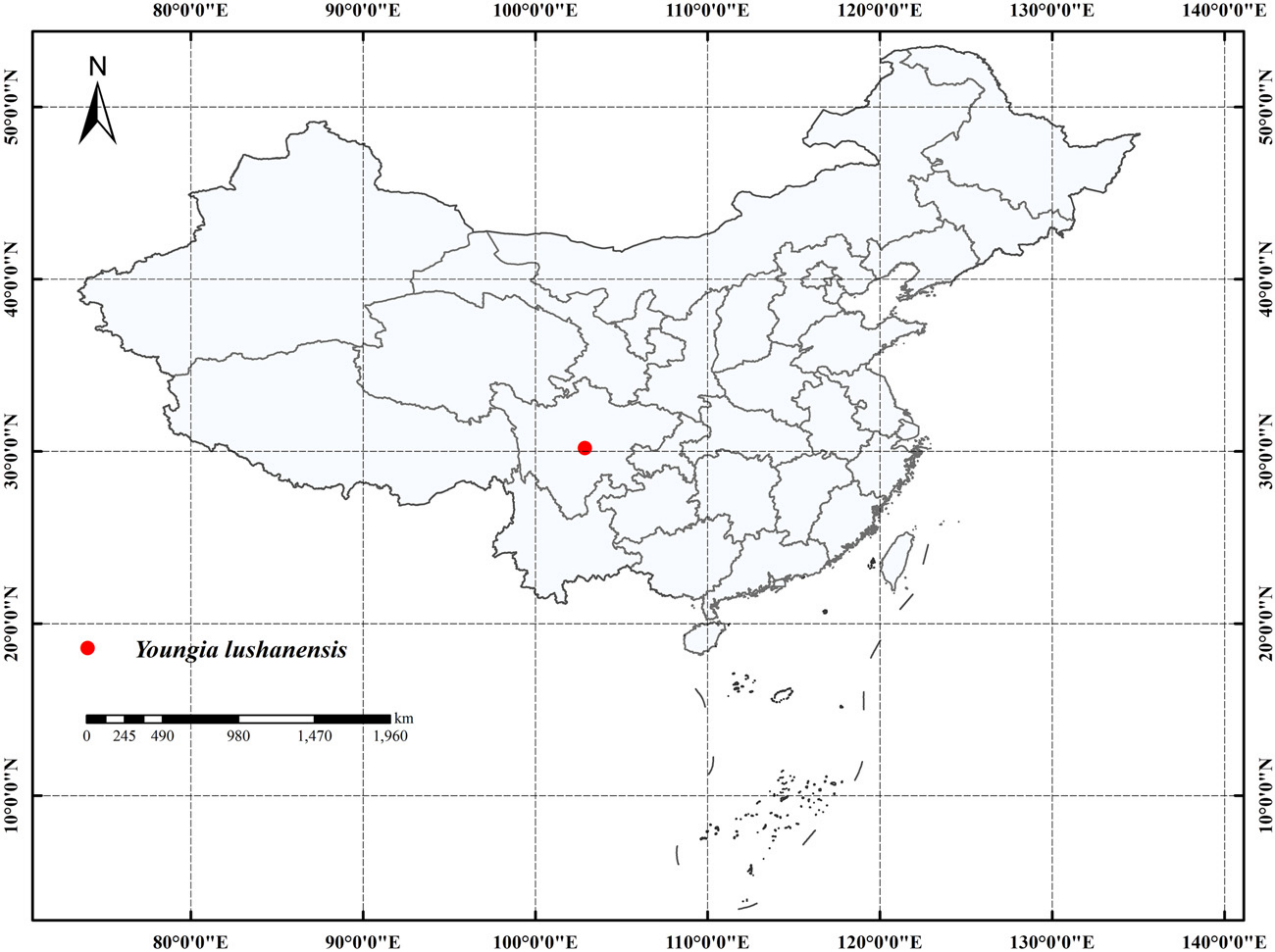
The distribution of *Youngia lushanensis*.

All the *Youngia* species were collected from China. The presumed new species *Y*. *lushanensis* were collected from Lushan County, Sichuan, Southwest of China (**Figure 1**). Lushan County is located at “Huaxi Rain Screen Belt” (Zhuang and Gao, 2002). This area is subtropical monsoon climate with an average annual precipitation of 1950-2000 milliliters, was famous for its high diversity (Sun et al., 2019).

### 2.3 DNA extraction, PCR amplification and sequencing

Total genomic DNA was isolated and cleaned with TIANGEN plant genomic DNA extraction kit (TIANGEN Biotech., Beijing, China). One nuclear region *(ITS*) was analyzed. The quality of DNA extraction was detected by gel electrophoresis methods. Electrophoresis was carried out on 0.8% agarose gel, and the gel with clear and bright target bands was recovered. The obtained DNA was stored in 4°Crefrigerators for DNA amplification. All amplifications were performed in 50 µl volumes containing 34 µl deionized sterile water, 3 µl of 25 mmol/L MgCl_2_ solution, 5 µl Taq reaction buffer, 4 µl of a 2.5 mmol/L dNTP solution, 1 µl of each primer at 10 p mol/mL, 1 µl (2.5 unit) Taq DNA polymerase (Tiangen), and 1 µl genomic DNA (20–100 ng). All amplifications were conducted on a DNA Engine Peltier Thermal Cycler (Bio-Rad Laboratories, USA). All the regions used the same thermal profiles, including a 5 min denaturation at 94°C followed by 33 cycles of 94°C for 45 s, 54°C for 1 min, and extension at 72°C for 1 min, with a final extension of 72°C for 7 min prior to holding at 12°C forever. The PCR products were separated by electrophoresis using 0.8–1.0% agarose gels and visualized with ethidium bromide. Amplified products were purified using E. Z. N. A. gel extraction kit (OMEGA, Biotech., USA). *ITS* primers its1 (White et al., 1990) and *itsb* (Blattner, 1999). All *ITS* fragment sequencing results of the samples were normal with single peak between 600-700 bp. The comparison results using Blast software in GenBank also indicate that the sequenced samples were the *ITS* gene, and then were used for phylogenic analysis.

### 2.4 Phylogenetic analysis

The sequences were aligned with Bioedit 7. 1.11 (Hall, 1999), followed by manual adjustment of the misaligned bases by the software. The gaps caused by mononucleotide repeat units were not considered in the phylogenetic analysis, because their homology can be highly uncertain (Kelchner, 2000). Prior to the model-based MP and BI approaches, the best fit model of nucleotide substitutions for the *ITS* sequence data was explored with the hierarchical likelihood ratio test as implemented in jModelTest2 (Darriba et al., 2012), available at https://groups.google.com/forum/#!forum/jmodeltest, which suggested TIMef+G as the best model. We used the aligned sequences to construct an ML phylogenetic tree was performed using RAxML-HPC2 (8.1.2) (Stamatakis, 2014), available at http://bioinformatics.oxfordjournals.org/content/early/2014/01/21/bioinformatics.btu033.abstract) with 1000 rapid bootstrap analyses followed by a search for the best scoring tree in one single run. BI was run using MrBayes v.3.1.2 (Ronquist et al., 2012). Bayesian tree topology was determined from two independent Markov chain Monte Carlo (MCMC) runs of four incrementally heated chains. Runs were performed for 10 million generations with sampling of trees every 1000 generations. When the log-likelihood scores were found to have stabilized, a consensus tree was calculated after discarding the first 25% of trees as burn-in.

## 3 Results

### 3.1 Morphological assessment

**Figure 2:**
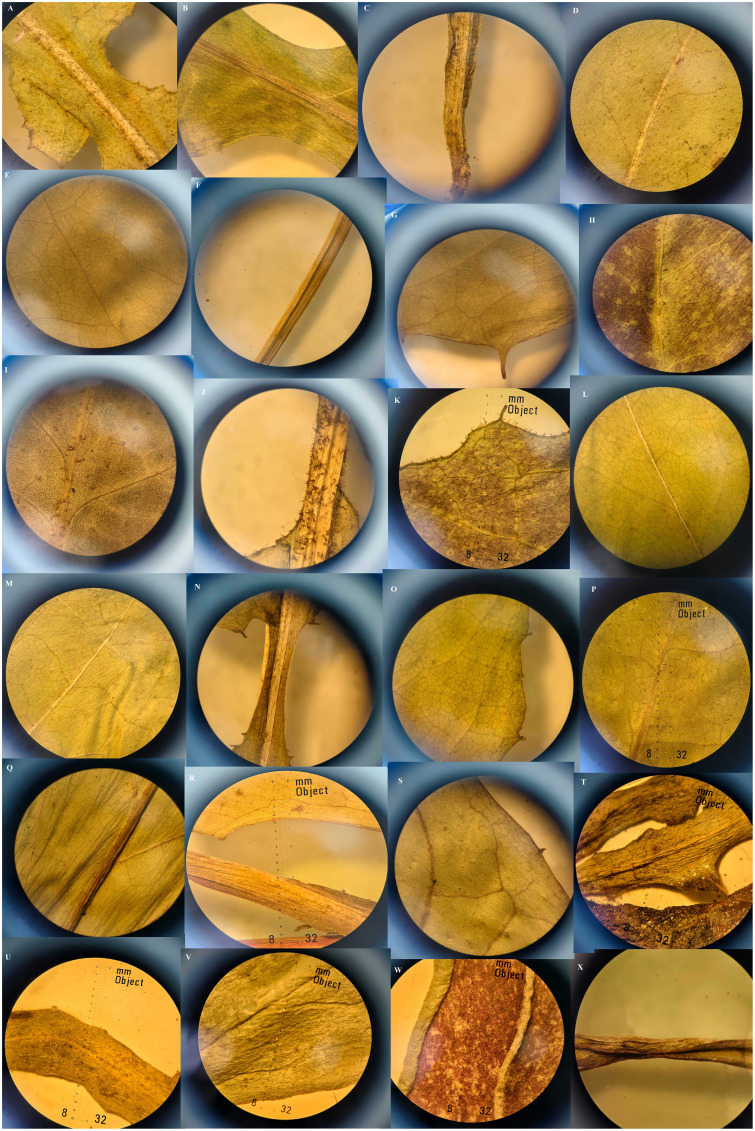
Indumentum of leaves, margin and petioles of *Youngia*. **(A–C)** *Y. Paleacea*, **(A)** Abaxial surface, **(B)** Ventral surface, **(C)** Petiole; **(D–G)** *Y. purpimea*, **(D)** Abaxial surface, **(E)** Ventral surface, **(F)** Petioles, **(G)** Margin; **(H–K)** *Y. szechuanica*, **(H)** Abaxial surface, **(I)** Ventral surface, **(J)** Petiole, **(K)** Margin, **(L–O)** *Y. heterophylla*, **(L)** Abaxial surface, **(M)** Ventral surface, **(N)** Petiole, **(O)** Margin; **(P–S)** *Y. cineripappa*, **(P)** Ventral surface, **(Q)** Abaxial surface, **(R)** Petiole, **(S)** Margin; **(T, U)** *Y. gracilipes*, **(T)** Abaxial surface and margin, **(U)** Ventral surface and margin; **(V–X)** *Y. lushanensis*, **(V)** Ventral surface & margin, **(W)** Abaxial surface, **(X)** Petiole.

**Figure 3:**
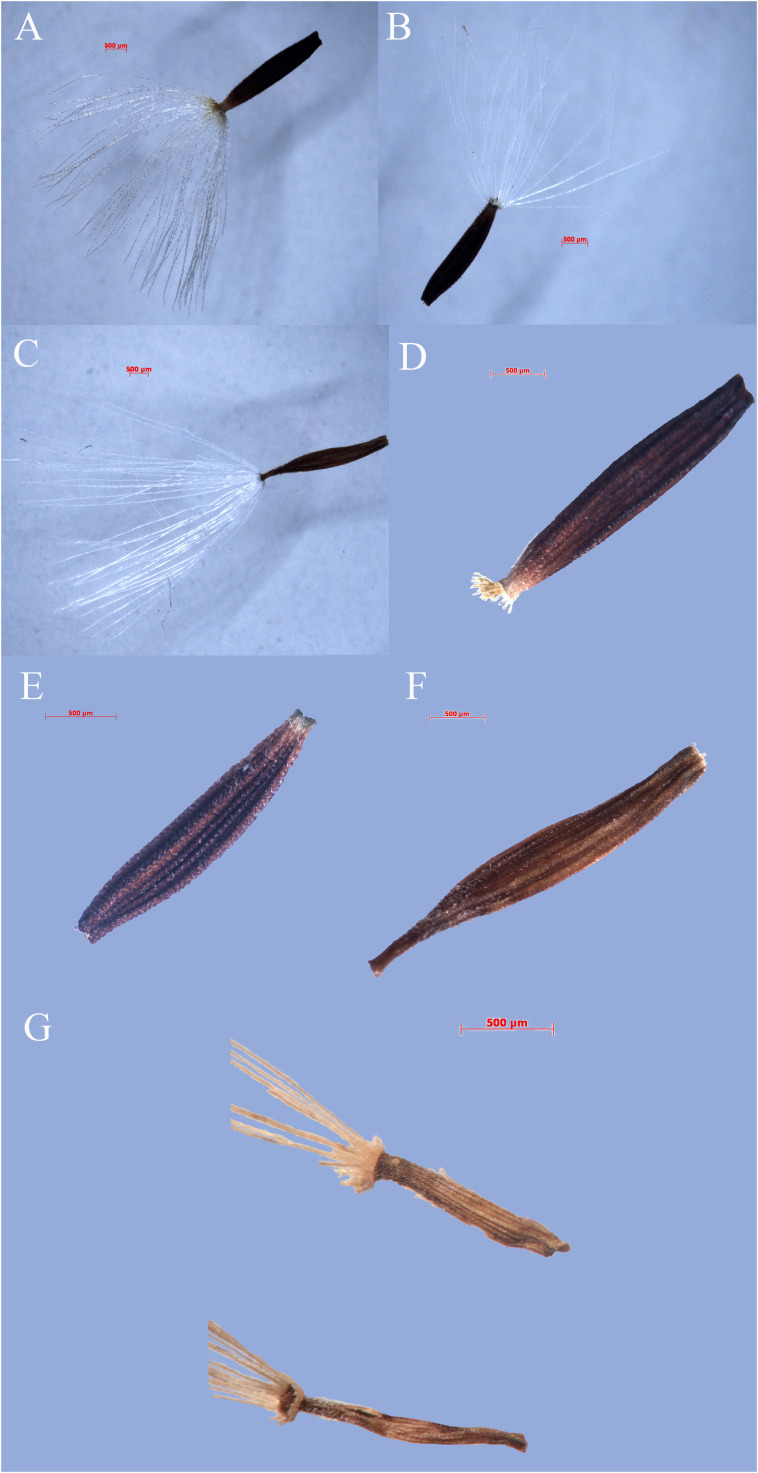
Fruits of *Youngia lushanensis* and related species. **(A, F)** *Y. paleacea*; **(B, E)** *Y. heterophylla*; **(C, D)** *Y. cineripappa*; **(G)** *Y. lushanensis*.

Qualitative characters comparison between *Youngia lushanensis*, *Y. szechuanica*, *Y. purpimea*, *Y. zhenyiana*, *Y. baoxingensis*, *Y. heterophylla*, *Y. gracilipes*, *Y. cineripappa*, and *Y. paleacea* were listed in **Table 2**. The inner phyllaries of three species including *Y. lushanensis*, *Y. paleacea* and *Y. baoxingensis* are crested with a narrow curved claw near tips. While the inner phyllaries of the other species are smooth and without coronal appendages. The leaf color of *Y. lushanensis*, *Y. szechuanica*, *Y. purpimea* and *Y. zhenyiana* is similar, with green surface green, purplish red abaxial surface, that of the rest is green on both surface. Indumentum on the surface of leaves and petioles varies among different species (**Figure 2**, **Table 2**). The leaves and petioles of *Y. szechuanica* have brown multicellular pubic hairs on both surfaces, those of *Y*. *paleacea* are glabrous or very sparsely white tomentose, those of *Y*. *heterophylla* & *Y. gracilipes*, are sparsely pubescent. And those of *Y*. *lushanensis* and the other surface are glabrous. The three species including *Y. lushanensis*, *Y. szechuanica* and *Y. purpimea* have yellowish pappus. The pappus of *Y. cineripappa* are grayish, that of *Y*. *baoxingensis* are brown, and the rest are white (**Figure 3**). The achenes of *Y*. *lushanensis* are yellowish brown, and those of the other compared species are dark brown or blackish brown. Quantitative characters comparison between the related species were listed in **Table 3**. T-test showed that the flores number per capitulum of the new species *Y. lushanensis* are significantly different from *Y. szechuanica*, *Y. purpimea*, *Y. zhenyiana*, *Y. baoxingensis*, *Y. gracilipes* and *Y. cineripappa* (**Table 4**). Number of florets per capitulum, number of capitula in the whole inflorescences, leaf length and plant height are significantly different between *Y. lushanensis* and *Y. baoxingensis*.

**Table 2:** Qualitative character comparison between *Youngia lushanensis, Youngia szechuanica, Youngia purpimea, Youngia zhenyiana, Youngia baoxingensis, Youngia heterophylla, Youngia gracilipes, Youngia cineripappa* and *Youngia paleacea*.

Characters	*Youngia lushanensis*	*Youngia szechuanica*	*Youngia purpimea*	*Youngia zhengyiana*	*Youngia paleacea*	*Youngia cineripappa*	*Youngia gracilipes*	*Youngia baoxingensis*	*Youngia heterophylla*
**Leaf attachment pattern**	Rosulate	Rosulate	Rosulate	Numerous basal leaves and cauline leaves	Numerous basal leaves and cauline leaves	Numerous basal leaves and cauline leaves	Rosulate	Numerous caudical leaves	Numerous basal leaves and cauline leaves
**Leaf texture**	Coriaceous	Thickly Papery	Coriaceous	Papery	Papery	Papery	Papery	Papery	Papery
**Leaf color**	Surface green, abaxial surface purplish red	Surface green, abaxial surface purplish red	Surface green, abaxial surface purplish red	Surface green, abaxial surface purplish red	Green	Green	Green	Green	Green
**Leaf shape**	Undivided, with concave midrib and lateral veins on the leaf surface, blade ovate	Pinnately deeply divided, blade oblanceolate.	Undivided, lateral veins not obvious on the leaf surface, blade ovate,	Lower leaves oblanceolate, lyrately pinnatisect, lateral lobes 1–2 pairs; upper leaves subulate	Basal and lower stem leaves oblanceolate, elliptic or narrowly elliptic, lateral lobes 2–7 pairs,	Basal leaves obovate to oblanceolate, lateral lobes 4–5 pairs; stem leaves elliptic to lanceolate,	Rosette leaves oblanceolate elliptic, or narrowly elliptic, pinnatifid to pinnatipartite, lateral lobes 3–6 pairs	Blade oblong, obvious reticular veins on the ventral surface,	Basal and lower stem leaves lyrately pinnatipartite or sometimes undivided
**Leaf margin**s	With inconspicuous short teeth	Serrated	With fine awn shaped teeth,	With conspicuous teeth	Entire or sinuate-dentate	Subentire to sinuate-dentate	Entire to weakly sinuate-dentate	Remotely denticulate to subentire	Entire or few serrate
**Leaf blades**	Glabrous	With brown multicellular pubic hairs on both surfaces	Glabrous	Glabrous.	Blade with brown hairs	Glabrous	Pubescent	Glabrous	Pubescent with short hairs
**Stems**	Glabrous, with rhizomatic rootstock	With densely brown or brown villus on the remaining petiole base	Brown cotton wool on base, with rhizomatic rootstock	Glabrous, erect	Glabrous or very sparsely white tomentose, erect	Glabrous, erect, rhizome horizontal or oblique, with fleshy fibrous roots, caudex short	Glabrous, caudex short	Glabrous, caudex short, with rhizomatic rootstock	Glabrous or sparsely hairy, erect
**Inner Phyllaries**	Crested with a narrow curved claw near tip	Smooth, without coronal appendages	Smooth, without coronal appendages	Smooth, without coronal appendages	Crested with a narrow curved claw near tip	Smooth, without coronal appendages	Smooth, without coronal appendages	Crested with a narrow curved claw near tip	Smooth, without coronal appendages
**Achenes**	Immature achenes light brown, mature achenes yellowish brown.	Dark purplish brown	Immature brown	Immature	Dark brown to blackish	Dark Brown	Black	Blackish brown	Blackish brown
**Pappus**	Yellowish-brown	Yellowish brown	yellowish-brown	White	White	Grayish	White	Brown	White

**Table 3:** Quantitative character comparison between *Youngia lushanensis*, *Y. szechuanica*, *Y. purpimea*, *Y. zhenyiana*, *Y. baoxingensis*, *Y. heterophylla*, *Y. gracilipes*, *Y. cineripappa* and *Y. paleacea*.

Species	Leaf length (cm)	Leaf width (cm)	Length of inner phyllaries (cm)	Number of Florets	Achenes length (cm)	Plant height (cm)	Number of capitula	Pappus length (cm)
*Youngia heterophylla* (A)	13.34 ± 6.80 (7~17)	4.26 ± 1.80 (2~8)	0.67 ± 0.12 (0.5~0.8)	14 ± 2.94 (11~25)	0.26 ± 2.46 (0.2~0.4)	32.55 ± 2.20 (38~100)	25.00 ± 2.50 (21~30)	0.30 ± 0.84 (0.3~0.5)
*Youngia gracilipes* (B)	4.5 ± 3.29 (3~14)	0.89 ± 0.56 (0.5~2.3)	0.78 ± 0.12 (0.5~1)	(14)	0.3 ± 0.09 (0.2~0.4)	6.73 ± 1.56 (1~23)	24.00 ± 4.03 (19~30)	0.6 ± 0.11 (0.5~0.7)
*Youngia szechuanica* (C)	15.04 ± 4.82 (10~23)	3.87 ± 1.30 (3~5)	0.59 ± 0.13 (0.5~0.7)	(4)	0.22 ± 0.05 (0.12~0.28)	26.18 ± 7.33(19~41)	47.00 ± 23.61 (23~75)	0.34 ± 0.03(0.3~0.4)
*Youngia cineripappa* (D)	13.65 ± 7.18(5~20)	4.06 ± 1.93 (2~6)	0.70 ± 0.22 (0.4~0.8)	(15)	0.26 ± 0.18 (0.2~0.4)	57.59 ± 43.48 (28~129)	15.00 ± 1.90 (13~18)	0.47 ± 0.14 (0.3~0.6)
*Youngia purpimea* (E)	16.57 ± 5.60 (12~21)	3.25 ± 1.32 (2~6)	0.53 ± 0.17 (0.2~0.7)	(5)	0.085 ± 0.020 (0.1)	30.72 ± 3.21 (27~35)	57.5 ± 0.71 (57~58)	0.45 ± 0.05(0.4~0.5)
*Youngia lushanensis* (F)	(8~10)	(1~3)	(0.7~0.8)	(5~9)	(0.4~0.6)	(30~32)	22.5 ± 4.5(18~27)	0.33 ± 0.08(0.25~0.4)
*Youngia zhenyiana* (G)	(15)	(5)	(0.5~0.7)	(5)	(0.3)	(15~30)	35 ± 9(26~44)	(0.4)
*Youngia baoxingensis* (H)	5 ± 2(3~7)	1.1 ± 0.03(0.8~1.4)	0.95 ± 0.05(0.9~1.0)	6.5 ± 0.5(6~7)	0.4 (0.4)	24 ± 6(18~30)	(18)	0.5 ± 0.1(0.4~0.6)
*Youngia paleacea* (I)	10.47 ± 3.27 (7~19)	2.25 ± 1.42(1~6)	1.02 ± 0.20 (0.8~1.4)	12.5 ± 3.5(9~16)	0.57 ± 0.26 (0.2)	25.9 ± 9.87 (18~66)	16.00 ± 1.60 (13~18)	0.65 ± 0.13 (0.5~0.8)

**Table 4:** T-test of the quantitative characters of *Youngia lushanensis* and related species.

Characters	F*A	F*B	F*C	F*D	F*E	F*G	F*H	F*I
**Length of inner phyllaries**	0.00^*^	0.44	0.01^*^	0.26	0.06	0.16	0.30	0.18
**Number of florets**	0.19	0.05^*^	0.02^*^	0.03	0.02^*^	0.50^*^	0.04^*^	0.28
**Achene length**	0.15	0.03^*^	0.01^*^	0.16	0.00^*^	0.02^*^	0.21	0.38
**Plant height**	0.28	0.00^*^	0.43	0.29	0.31	0.21	0.18	0.35
**Number of capitula**	0.18	0.00^*^	0.19	0.02	0.08	0.08	0.09	0.12
**Pappus length**	0.50	0.006^*^	0.46	0.17	0.15	0.21	0.15	0.10
**Leaf Length**	0.23	0.00^*^	0.07	0.15	0.05	0.07	0.07	0.28
**Leaf width**	0.04	0.37	0.04	0.01	0.12	0.05	0.24	0.19

^*^ indicate Significance at 0.05 of T Test. A, B, C, D, E, F, G, H and I is the alternative name of the species named in **Table 3**.

### 3.2 Phylogenetic tree

**Figure 4:**
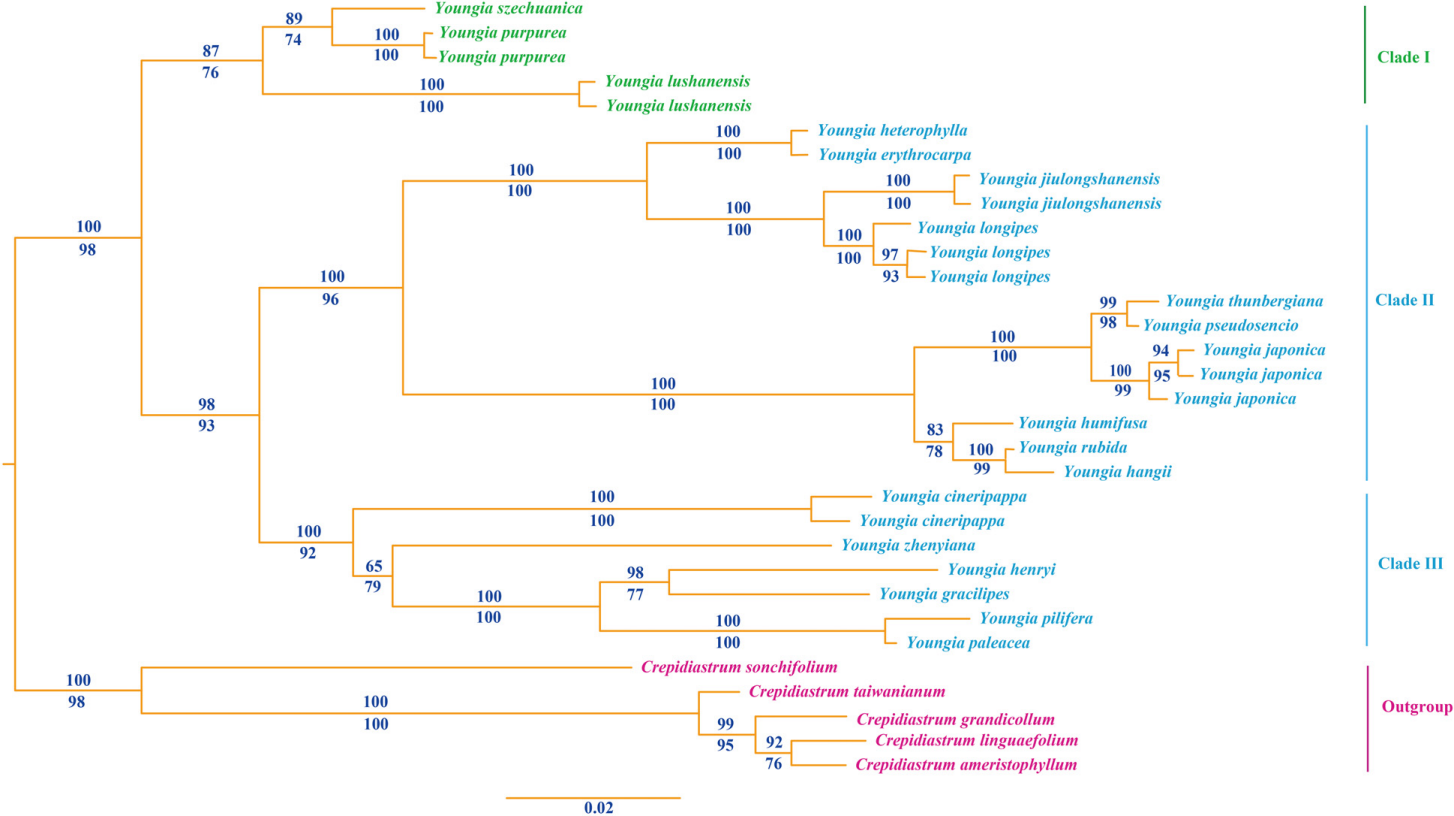
Bayesian consensus tree of the new species and related species of *Youngia* based on *ITS* sequences. Numbers above branches indicate Bayesian posterior probability [PP], numbers below branches are ML bootstrap [LP]. The new species and its sister groups are shown in green font.

From the Bayesian tree (**Figure 4**), the species of *Youngia* formed a monophyletic group with strong support (PP= 1.0, LP = 98), which demonstrated that the presumed new species belonged to the genus. It revealed that the samples were clustered into three major clades of *Youngia* species (**Figure 1**). Clade I is the base group includes *Y*. *szechuanica* of “sect. *Hieraciella*”, *Y*. *lushanensis*, *Y. purpime*a, which is the sister group of Clade II and Clade III. Clade II includes “sect. *Euyoungia*”. Clade III include species of “sect. *Mesomeris*” and species of “sect. *Desiphylum”.* The presumed new species was nested within the Clade I, was closely related to *Y. purpime*a and *Y. szechuanica* (E. S. Soderberg) S. Y. Hu.

## 4 Discussion

**Figure 5:**
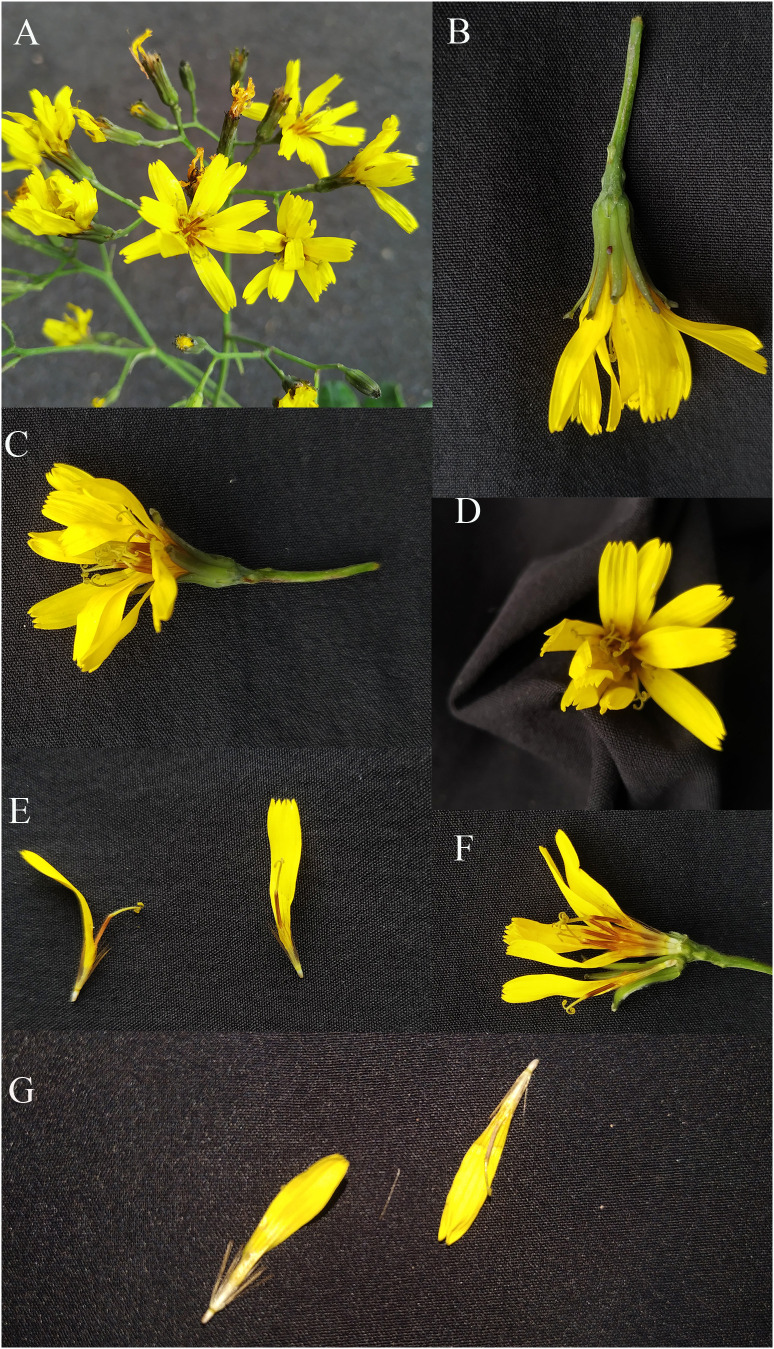
Flowers & Fruits of *Youngia lushanensis*. **(A)** Inflorescence. **(B)**. Capitulum & phyllaries. **(C–F)**. Florets. **(G)** Pappus, immature achenes and ligulate flowers.

Both phylogeny analysis based on *ITS* and morphology study showed that the presumed new species *Youngia lushanensis* was closely related with *Y. purpimea* and *Y. szechuanica*. However, it can be easily recognized by its crested inner phyllaries and 9–11 florets in each capitulum (**Figure 5A, C, D**)*. Youngia lushanensis* is similar to *Y. purpimea* (Peng et al., 2015) for its leaves are undivided, glabrous and purplish red on the back. While in *Y*. *lushanensis*, the middle and lateral veins are concave in the leaf surface and margins with inconspicuous short teeth, caudex is glabrous, each capitulum contains 9–11 florets (vs 5 florets in *Y*. *purpimea*), and inner phyllaries are crested (**Table 2**; **Figure 5B**). *Youngia lushanensis* is similar to *Y. szechuanica* (Hu, 1969), with fewer florets in the capitula, comparing with the other species in the *Youngia* genus. But the leaves of the latter are pinnately deeply divided, both surfaces covered with brown ruffled multicellular stub hairs, and the petiole base is covered with dense brown villus (**Table 2**). *Youngia lushanensis* is also similar to *Y*. *baoxingensis* (Chen, 2018) with crested inner phyllaries. But is distinctive from the later for ovate leaves and 9–11 florets (vs 6–7 florets in *Y*. *baoxingensis*) (**Figure 2**, **Figure 3**). The habitat of *Y. lushanensis* is similar to *Y*. *purpimea*, growing in the rocky slopes with flowing water in lower altitude 780–795 m*. Youngia purpimea* were found in the rocky slopes with flowing water in Xuyong and Gulin County, Southwest of Sichuan, with the altitude from 650–1200 m, according to the investigation of Peng et al. (2014), and specimen records [*S.N. 890* (SM); *S.N. 0221* (SM), *S.N. 77-503* (SM)]. *Youngia szechuanica* was also found in moist stone slopes in Hejiang County of Sichuan Province, Qijiang and Nanchuan County of Chongqing City, with the altitude from 650–1700 m, according to in our field survey and specimen records [*Zhang 1030* (KUN), *Z.Y. Liu 183148*(PE), *W.P. Fang* (PE); *J.H. Xiong & Z.L. Zhou 92210* (PE), *J.H. Xiong & Z.L. Zhou 91831(*PE); *Gu-0821* (SM); *J.H. Xiong & B.Q. Li 95266* (SM); *J.H. Xiong & B.Q. Li 95060* (SM); *Z.C. Zhong 01430* (SM)]. The three species formed a basal clade of the genus *Youngia* (“sect. *Hieraciella*”). While *Y. baoxingensis* grows on grassy slopes at the margins of mixed forests at elevations of 2950–3200 m (Chen, 2018). Changes in environmental moisture conditions may lead to the rapid diversification of the genus *Youngia*, as observed in the critical genera linked to peculiar environment condition (Cheng et al., 2022; Perrino et al., 2022; Calvo et al., 2020; Perrino et al., 2023). Due to the lack of molecular materials, this could not be included in the phylogenetic tree. However, based on morphology and habitat, it could belong to the species of “sect. *Mesomeris*”, which is consistent with viewpoint of Chen (2018). Strangely, although only five florets per capitulum in *Y. zhenyiana*, which is similar to the *Y. szechuanica* and *Y. purpimea* of the “sect. *Hieraciella*”. But the pappus of *Y. zhenyiana is white* and leaf texture is papery, which make it is significantly different from the “sect. *Hieraciella”*. Phylogeny tree based on *ITS* suggests that it belongs to another group (“sect. *Mesomeris*”) (**Figure 4**). The presumed species, *Y. purpimea* and *Y. szechuanica* could be easily distinguished from the other species of *Youngia* by their special leaf texture, fewer florets and yellowish brown pappus. We will provide classification and retrieval keys for relevant species in the flowing paraphrases.

### 4.1 Key to *Youngia lushanensis* and related species

1a Perennial, florets 5–11 per capitulum, pappus yellowish, leaves slightly leathery or thicker, growing in wet slopes with flowing water…………………………………………2
2a Florets 5 per capitulum, inner phyllaries glabrous, the main stem base covered with dense brown or brown long soft hairs…………………………………………………………3
3a Leaves, lyrately pinnatilobate, pinnatipartite, or pinnatisect, both faces pubescent with brown multicellular crinkled hairs………………………………...…*Youngia szechuanica*
3b Leaves undivided, margin sparsely and shallowly sinuate-dentate with long-acuminate teeth…*Youngia purpimea*
2b Florets 9–11 per capitulum, inner phyllaries crested, stem base glabrous…………………………..*Youngia lushanensis*
1b Perennial or annual, florets5–16 per capitulum, grayish or white, usually growing in drier habitat……………………4
4a Annual or biennial…………………………………………5
5a Biennial, achenes dark brown……….*Youngia heterophylla*
5b Annual, achene reddish brown………….*Youngia japonica*
4b Perennial……………………………………………………6
6a Pappus grayish, leaves undivided to pinnatifid or lyrately pinnatifid……………………………...*Youngia cineripappa*
6b Pappus white or brown, leaves undivided to pinnatifid or lyrately pinnatifid…………………………………………..7
7a Florets 5, Papppus white………………*Youngia zhenyiana*
7b Florets more than six, pappus white or brown……………8
8a 1–23 cm tall, rosulate, subacaulescent or dwarf, caudex short …………………………………………....*Youngia gracilipes*
8b 18–66 cm tall, rosulate, caudex long, stem erect …….………………………………………*Youngia paleacea*

## 5 Taxonomy

**Figure 6:**
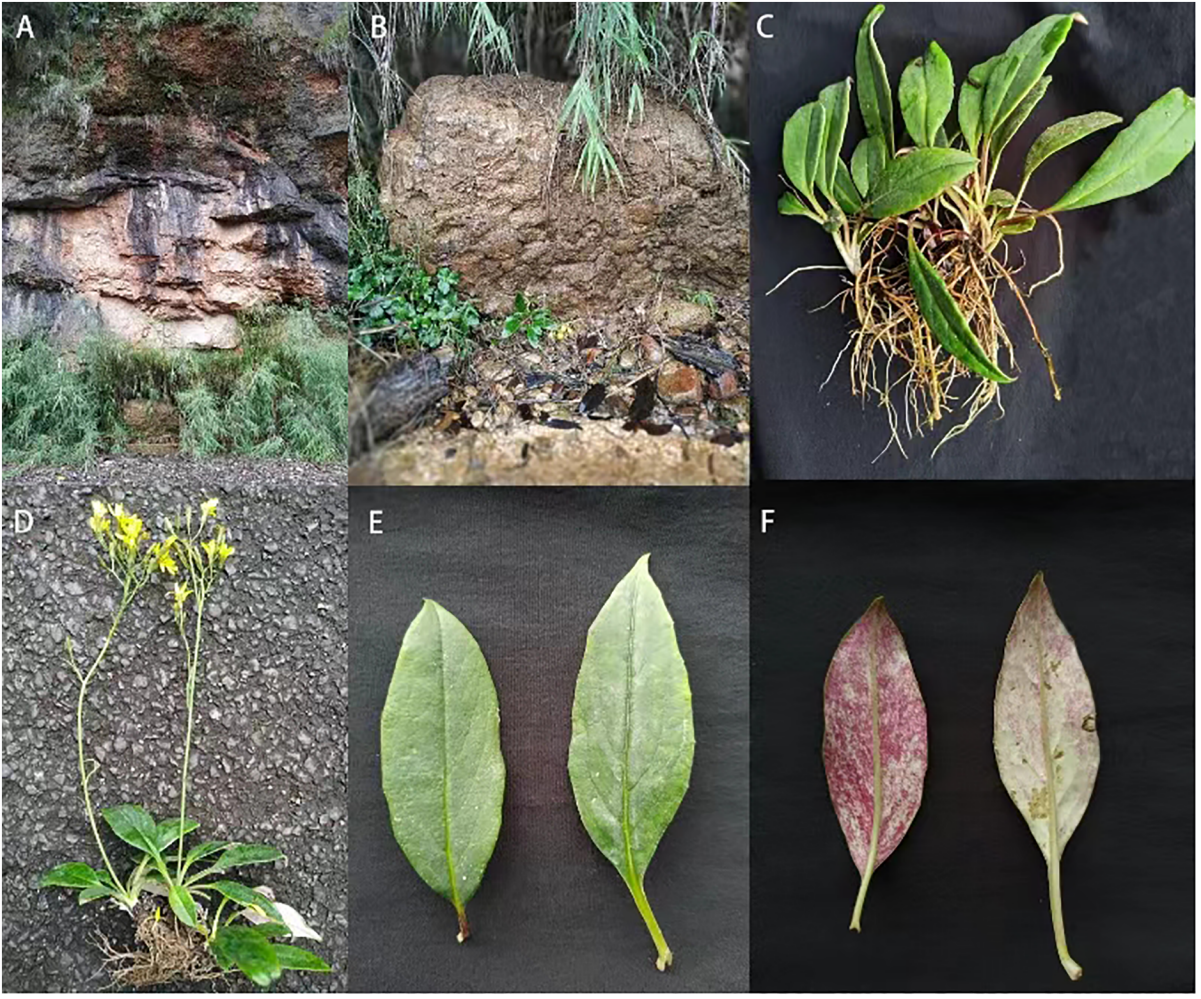
*Youngia lushanensis* X.H. Xiong, Y.L. Peng, X.M. Pu, L.J. Cheng & W.B. Ju sp. *nov.* **(A, B)** Habitat. **(C, D, G)** Whole living plants. **(E)**. Leaf ventral surface. **(F)**. Leaf dorsal surface. **(H)**. Inflorescence.

*Youngia lushanensis* X.H. Xiong, Y.L. Peng, X.M. Pu, L.J. Cheng & W.B. Ju sp. *nov.* (**Figures 5**, **6**).

Diagnosis: —The new species most closely resembles *Y. szechuanica* and *Y. purpimea*, but differs in having thick and leathery leaves, with concave midrib and lateral veins on the ventral surface, margins sparsely dentate, and being glabrous on both surfaces (**Figures 6C–H**), and 9–11 florets per capitulum, inner phyllaries crested with a narrow curved claw near tip.

Type: —CHINA. Sichuan Province: Luyang Street, Lushan County, 788.7 m, 2 June 2023. X.H. Xiong 20230602-1 (holotype, CDBI)! (Paratype, X.H. Xiong 20230602-2 CDBI).

Herbs, 30–32 cm tall, perennial, often rosulate, with rhizomatic rootstock. Fibrous roots vertically extended or skewed. Caudex glabrous. Stem upright, solitary, thick and robust, glabrous. Caudical leaves 4–5, thick and leathery, narrowly ovate, 8–10 cm long and 1–3 cm wide, glabrous, green on the ventral surface and purple red on abaxial surface, the midrib of the leaf blade concave on the surface, with 3–4 pairs lateral veins that arise from the midrib of the blade, margin entire or sparsely dentate, apex acuminate, base attenuate, petiole 2–2.5 cm long, petiole base smooth and glabrous. Cauline leaves 1–2, linear, bract-like. Involucre cylindrical, green. Synflorescence corymbiform; capitula 18–27; peduncles slender, 3–4 cm, glabrous. Outer phyllaries 5, 0.9 mm long, ovate, inner phyllaries 9–10, 8–9 mm long, lanceolate, crested with a narrow curved claw near tip. Florets 9–11, ligule, yellow, 10–15 mm long, 5-toothed. Anther tube yellow to brown, ca. 3 mm long; Style branches yellow, 1.0 mm long. Immature achenes light brown, fusiform, 2–3 mm, mature achenes 4-6 mm, with six uneven ribs, apex slightly attenuate, with small prickly hairs. Pappus, yellowish-brown, 2.5–4 mm long (**Figures 5E–G**).

Etymology: —”Lushan-” refers to the locality of the new species; “ -ensis” is an artificial ending.

Distribution and habitat: —This species was only found in the type locality, i.e., 30.1752 N, 102. 8705 E., Luyang Street, Lushan County, Sichuan Province, at elevations between 789–795 m. A total of 40-55 individual were found in this only population, growing on the slopes with flowing water, at the edge of mixed evergreen and deciduous broad-leaved forest, according to our field survey. This species grows on thin rocky swamp soil (**Figure 6A, B**).

Phenology: —Flowering and fruiting from May to July.

## Data Availability

The datasets presented in this study can be found in online repositories. The names of the repository/repositories and accession number(s) can be found below: https://www.ncbi.nlm.nih.gov/genbank/, PP785561, PP784683, PP784685, PP789654, PP785562, PP785563, PP785560.

## Supplementary material

Supplementary Material S1 Taxon name, voucher information for morphology studies ***Youngia baoxingensis***, *Y.S. Chen 7280* (PE); ***Youngia lushanensis***, *X.H. Xiong* 20230602-2, *X.H. Xiong*20230602-1 (CDBI); ***Youngia heterophylla***, *Y. L. Peng*, *Y.J. Li & H.L. Zhou SE03311* (CDBI), *Y. L. Peng*, *Y.J. Li & H.L. Zhou SE3341*(CDBI), X.F. Gao, Y.L. Peng, B. Xu & X. Zeng 11996 (CDBI), *Y.S. Che & Z.H. Wang 9102*(PE), *PE-Gulin Expedition 023*(PE), *Li Heng* et al. *24217*(PE), *Z.Y. Liu 17241*(PE), *K.J. Guan, J.W. Wang &C.L. Li* 596(PE), *G.F.* Li *61926*(PE), *Wuning Expedition 1029* (KUN), *X.F. Gao, Y.D. Gao & W. B. Ju HGX11476* (CDBI), *Y.L. Peng 102* (CDBI), *X.F. Gao*, *Y. L. Peng, B. Xu & X. Zheng 11612* (CDBI); *C.H. Li 893* (NAS); ***Youngia cineripappa***, *ZZ09097* (KUN), *ZZ09041* (KUN), Z.Z. *Zhu & W.B. Ju714*(CDBI), *Y. L. Peng 360*(CDBI), *Z.Q. Fang 836*(CDBI), *J.J. Zhou & H. Zhoui*1406 12008 (CSFI), *A. Liu, Y.K. Gong & C.Z. Huang* (CSFI), *Y.S. Chen* 7317(PE), *T.T. YU 6167* (KUN), *Henry A.11997* (E); *Y.S. Chen & Z.H. Wang* 9048(PE), *Y.S. Chen & Z.H. Wang* 9048 (PE); ***Youngia gracilipes***, *Boufford & al. 34392* (A, KUN), *Y.T. Zhang & K. Y. Lang 2338* (PE), *Xiang Expedition 3992* (PE), X.F. Gao, Z.M. Zhu, W.B. Ju & W.T. Jin 14783 (CDBI), *J.S. Yang 380* (KUN), *Biology Institute Expedition 3992*(CDBI), *S. Chen & Z.H. Wang 9321* (PE), *Y.S. Chen & Z.H. Wang 9358*(PE), *Y.S. Chen 9499A*(PE), *J.S. Ying & D.Y. Hong 651229(*PE); ***Youngia paleacea***, *Y. L. Peng*, *Y.J. Li & H.L. Zhou SE03326* (CDBI), Y. L. Peng, *Y.J. Li & H.L. Zhou SE03341* (CDBI), *Boufford & al. 34500* (*A*, KUN), *D.E. Boufford* et al. *43368* (PE); *Boufford & al.38233* (PE), *FLPH Tibet Expedition 12-1502* (PE), *FLPH Tibet Expedition 12-0587A (*PE), *Tibet Expedition 14959* (PE), *Y.S. Chen 7541* (PE), *K. Iwasuki* et al. (KUN), *L. Peng pl2011082605-3* (CDBI), *Y.L. Peng & L.J. Tong 1309*(CDBI); ***Youngia zhenyiana***, *Deng 2945* (KUN); ***Youngia purpimea***, *11571 (*CDBI), *Y. L. Peng*, *Y.J. Li & H.L. Zhou SE02106* (CDBI), *S.N. 890* (SM); *S.N. 0221* (SM), *S.N. 77-503* (SM); ***Youngia szechuanica***, *Y. L. Peng*, *Y.J. Li & H.L. Zhou SE02557* (CDBI), *Zhang 1030* (KUN), *Z.Y. Liu 183148*(PE), *W.P. Fang* (PE); *J.H. Xiong & Z.L. Zhou 92210* (PE), *J.H. Xiong & Z.L. Zhou 91831(*PE); *Gu-0821* (SM); *J.H. Xiong & B.Q. Li 95266* (SM); *J.H. Xiong & B.Q. Li 95060* (SM); *Z.C. Zhong 01430* (SM).
