# Supplementary material for: Youngia lushanensis, a new species of Asteraceae from Sichuan, China, inferred from morphology and nrITS phylogeny
Source: Front Plant Sci. 2024 Dec 4;15:1453759. doi: 10.3389/fpls.2024.1453759 (PMC11653769; doi:10.3389/fpls.2024.1453759)
Supplement: Supplementary Material S1 — Taxon name, voucher information for morphology studies Youngia baoxingensis , Y.S. Chen 7280 (PE); Youngia lushanensis , X.H. Xiong 20230602-2, X.H. Xiong20230602-1 (CDBI); Youngia heterophylla , Y. L. Peng, Y.J. Li & H.L. Zhou SE03311 (CDBI), Y. L. Peng, Y.J. Li & H.L. Zhou SE3341(CDBI), X.F. Gao, Y.L. Peng, B. Xu & X. Zeng 11996 (CDBI), Y.S. Che & Z.H. Wang 9102(PE), PE-Gulin Expedition 023(PE), Li Heng et al. 24217(PE), Z.Y. Liu 17241(PE), K.J. Guan, J.W. Wang &C.L. Li 596(PE), G.F. Li 61926(PE), Wuning Expedition 1029 (KUN), X.F. Gao, Y.D. Gao & W. B. Ju HGX11476 (CDBI), Y.L. Peng 102 (CDBI), X.F. Gao, Y. L. Peng, B. Xu & X. Zheng 11612 (CDBI); C.H. Li 893 (NAS); Youngia cineripappa , ZZ09097 (KUN), ZZ09041 (KUN), Z.Z. Zhu & W.B. Ju714(CDBI), Y. L. Peng 360(CDBI), Z.Q. Fang 836(CDBI), J.J. Zhou & H. Zhoui1406 12008 (CSFI), A. Liu, Y.K. Gong & C.Z. Huang (CSFI), Y.S. Chen 7317(PE), T.T. YU 6167 (KUN), Henry A.11997 (E); Y.S. Chen & Z.H. Wang 9048(PE), Y.S. Chen & Z.H. Wang 9048 (PE); Youngia gracilipes , Boufford & al. 34392 (A, KUN), Y.T. Zhang & K. Y. Lang 2338 (PE), Xiang Expedition 3992 (PE), X.F. Gao, Z.M. Zhu, W.B. Ju & W.T. Jin 14783 (CDBI), J.S. Yang 380 (KUN), Biology Institute Expedition 3992(CDBI), S. Chen & Z.H. Wang 9321 (PE), Y.S. Chen & Z.H. Wang 9358(PE), Y.S. Chen 9499A(PE), J.S. Ying & D.Y. Hong 651229(PE); Youngia paleacea , Y. L. Peng, Y.J. Li & H.L. Zhou SE03326 (CDBI), Y. L. Peng, Y.J. Li & H.L. Zhou SE03341 (CDBI), Boufford & al. 34500 (A, KUN), D.E. Boufford et al. 43368 (PE); Boufford & al.38233 (PE), FLPH Tibet Expedition 12-1502 (PE), FLPH Tibet Expedition 12-0587A (PE), Tibet Expedition 14959 (PE), Y.S. Chen 7541 (PE), K. Iwasuki et al. (KUN), L. Peng pl2011082605-3 (CDBI), Y.L. Peng & L.J. Tong 1309(CDBI); Youngia zhenyiana , Deng 2945 (KUN); Youngia purpimea , 11571 (CDBI), Y. L. Peng, Y.J. Li & H.L. Zhou SE02106 (CDBI), S.N. 890 (SM); S.N. 0221 (SM), S.N. 77-503 (SM); Youngia szechuanica , Y. L. Peng, Y.J. Li & H.L. Zhou SE02557 (CD [file DataSheet1.docx]

**Appendix S1**. Taxon name, voucher information for morphology studies

***Youngia baoxingensis***, *Y.S. Chen 7280* (PE); ***Youngia lushanensis***, *X.H. Xiong* 20230602-2, *X.H. Xiong*20230602-1 (CDBI); ***Youngia heterophylla***, *X.F. Gao, Y.L. Peng, B. Xu & X. Zeng* *11996* (CDBI), *Y.S. Che & Z.H. Wang 9102*（PE), *PE-Gulin Expedition 023*（PE), *Li Heng et al. 24217*（PE), *Z.Y*. *Liu 17241*（PE), *K.J*. *Guan, J.W. Wang &C.L. Li* 596（PE), *G.F*. Li *61926*（PE), *Wuning Expedition 1029* (KUN), *X.F. Gao, Y.D. Gao & W. B. Ju HGX11476* (CDBI), *Y.L. Peng 102* (CDBI), *X.F. Gao*, *Y. L. Peng, B. Xu & X. Zheng 11612* (CDBI); *C.H.* *Li 893* (NAS); ***Youngia cineripappa***, *ZZ09097* (KUN), *ZZ09041* (KUN), Z.Z. *Zhu & W.B. Ju714*（CDBI), *Y. L. Peng 360*（CDBI), *Z.Q. Fang 836*（CDBI), *J.J. Zhou & H. Zhoui*1406 12008 (CSFI), *A.* *Liu, Y.K. Gong & C.Z. Huang* (CSFI), *Y.S. Chen* 7317（PE), *T.T. YU 6167* (KUN), *Henry A.11997* (E); *Y.S. Chen & Z.H. Wang* 9048（PE), *Y.S. Chen & Z.H. Wang* 9048 (PE); *Y****oungia gracilipes***, *Boufford & al. 34392* (A, KUN), *Y.T. Zhang & K. Y. Lang 2338* (PE), *Xiang Expedition 3992* (PE), *X.F. Gao*, *Z.M. Zhu*, *W.B. Ju & W.T. Jin 14783* (CDBI), *J.S. Yang 380* (KUN), *Biology Institute Expedition 3992*（CDBI), *S. Chen & Z.H. Wang 9321* (PE), *Y.S. Chen & Z.H. Wang 9358*（PE), *Y.S. Chen 9499A*（PE), *J.S. Ying & D.Y. Hong 651229（*PE); ***Youngia paleacea***, *Boufford & al. 34500* (*A,* KUN)*，D.E. Boufford et al. 43368* (PE); *Boufford & al.38233* (PE), *FLPH Tibet Expedition 12-1502* (PE), *FLPH Tibet Expedition 12-0587A (*PE), *Tibet Expedition 14959* (PE), *Y.S. Chen 7541* (PE), *K. Iwasuki et al.* (KUN), *L. Peng pl2011082605-3* (CDBI), *Y.L. Peng & L.J. Tong 1309*（CDBI); *Y****oungia zhenyiana***, *Deng 2945* (KUN); ***Youngia purpimea***, *11571 (*CDBI), *S.N. 890* (SM); *S.N. 0221* (SM), *S.N. 77-503* (SM); ***Youngia szechuanica***, *Zhang 1030* (KUN), *Z.Y. Liu 183148*（PE), *W.P. Fang* (PE); *J.H. Xiong & Z.L. Zhou 92210* (PE), *J.H. Xiong & Z.L. Zhou 91831（*PE); *Gu-0821* (SM); *J.H. Xiong & B.Q. Li 95266* (SM); *J.H. Xiong & B.Q. Li 95060* (SM); *Z.C. Zhong 01430* (SM).
